# Human cold habituation: Physiology, timeline, and modifiers

**DOI:** 10.1080/23328940.2021.1903145

**Published:** 2021-05-25

**Authors:** Beau R. Yurkevicius, Billie K. Alba, Afton D. Seeley, John W. Castellani

**Affiliations:** a Thermal and Mountain Medicine Division, US Army Research Institute of Environmental Medicine, Natick, MA, USA; b Oak Ridge Institute of Science and Education, Belcamp, MD, USA

**Keywords:** Adaptation, shivering, vasoconstriction, skin temperature, cold air exposure, cold water immersion, thermoregulation, cold shock response

## Abstract

Habituation is an adaptation seen in many organisms, defined by a reduction in the response to repeated stimuli. Evolutionarily, habituation is thought to benefit the organism by allowing conservation of metabolic resources otherwise spent on sub-lethal provocations including repeated cold exposure. Hypermetabolic and/or insulative adaptations may occur after prolonged and severe cold exposures, resulting in enhanced cold defense mechanisms such as increased thermogenesis and peripheral vasoconstriction, respectively. Habituation occurs prior to these adaptations in response to short duration mild cold exposures, and, perhaps counterintuitively, elicits a reduction in cold defense mechanisms demonstrated through higher skin temperatures, attenuated shivering, and reduced cold sensations. These habituated responses likely serve to preserve peripheral tissue temperature and conserve energy during non-life threatening cold stress. The purpose of this review is to define habituation in general terms, present evidence for the response in non-human species, and provide an up-to-date, critical examination of past studies and the potential physiological mechanisms underlying human cold habituation. Our aim is to stimulate interest in this area of study and promote further experiments to understand this physiological adaptation.

## Introduction

Human beings, when exposed to cold environments, exhibit a range of adaptations that are dependent on the number, duration, and severity of cold exposures. The primary adaptations that have been documented include a) hypermetabolic, b) insulative, and c) habituated responses [1,2]. A hypermetabolic adaptation has traditionally been defined as an enhancement in metabolic heat production, often through increased shivering thermogenesis, though the data supporting this type of response are sparse [3]. Recent data suggest that non-shivering thermogenesis may be a part of this increased heat production. An insulative adaptation is characterized by a greater degree of cutaneous vasoconstriction, resulting in lower skin temperatures and a reduction in peripheral heat loss. Insulative and hypermetabolic adaptations to the cold are not frequently observed in modern society as humans today typically engage in behavioral thermoregulation aided by the development of modern clothing, heated buildings, and vehicles that allow for the maintenance of thermoneutral microenvironments and comfort in the winter months. Interested readers are referred to excellent reviews for additional information on hypermetabolic and insulative adaptations to chronic cold stress [1,2,4,5].

The specific focus of this review is cold habituation, the most prevalent cold adaptation in modern society due to a comfort-driven reluctance to expose more than small body segments to the cold during winter months. Habituated responses to cold exposure are typically observed in environments that elicit cutaneous cooling, but no decline in core temperature, provoked either by brief or mild whole-body or localized cold exposures [1]. Cold habituation is marked by and defined as an attenuated (i.e., a smaller increase in) cutaneous vasoconstriction and/or metabolic heat production. Other physiological changes with habituation include a blunted blood pressure (BP) response and decreased catecholamine release [6–8]. Cold habituation also results in a reduced sensation of cold, such as when a mild day (10–15°C) seems far warmer in Spring versus Autumn.

The following are definitions of the general terms used in this review, as defined by the International Union of Physiological Sciences [9]. The term adaptation is used to describe “changes that reduce the physiological strain produced by stressful components of the total environment”. The terms acclimation and acclimatization are often used interchangeably to refer to any adaptive change which occurs due to prolonged or repeated exposure to a stressful environment, and which reduces the strain or enhances endurance of strain in that environment. The terms differ slightly in that acclimation refers to experimentally driven or lab-based exposures, while acclimatization refers to natural exposures due to climate, season, or location. Habituation is defined as a “reduction of responses to or perception of a repeated stimulation.” In the context of this review, adaptation will be used as a general term, acclimation and acclimatization will be used to differentiate exposure type within the profiled studies, and habituation will be used to describe a reduction in the typical responses observed during acute cold exposures.

The purpose of this review is to generally define habituation, present evidence for the response in non-human species, and, most importantly, provide an up-to-date, critical examination of past studies and the potential physiological mechanisms underlying human cold habituation. Exploring such adaptations to cold environments may be important for specific populations including the military, outdoor workers, and athletes. Our aim is to stimulate interest in this area of study and promote further experimentation to broaden our physiological understanding of cold habituation.

## General habituation

Traditionally, habituation has referred to a diminution of nervous system responses to repeated stimuli. Repetition of a sensed stimulus often results in a reduced autonomic response and blunting of efferent output of the central nervous system. This autonomic blunting results from a decreased perception of the repeated stimulus, which is thought to allow the organism to filter out irrelevant input to focus on more important stimuli. Habituation has also been described as a basic “learning” or memory process, one where the organism naturally learns what is not harmful in order to increase survivability [10]. It is likely that many habituation responses are evolutionarily preserved as a protective mechanism to reduce physiological burden. Habituation to stress, particularly activators of the hypothalamic-pituitary axis (HPA) and sympathetic nervous system (SNS) (e.g., restraint, novel environment, water immersion, noise, and psychosocial stress), are consistently observed in both animals and humans [11,12]. Activation of stress systems are metabolically costly and can be deleterious for health and survival if overactive; thus, habituation to repeated, homotypic stressors may conserve energy and resources by attenuating responses to non-life threatening stressors while maintaining responsiveness to unique stimuli [12].

An early description of habituation came from Sokolov in 1963 [13], in which he described a diminished orientation reflex after repeated complex stimuli and an instantaneous response recovery upon altering the stimulus. Thompson and Spencer [14] expanded upon Sokolov’s work to further describe this phenomenon, which included a set of characteristics that were common among previous studies examining habituated responses. Thompson and Spencer defined habituation as an exponential decline in the response to repeated stimuli which recovers over time if the stimulus is removed. They postulated that if the stimulus is repeatedly applied and removed, habituation takes place at a faster rate during each series, and often responds faster to weaker and more frequent stimuli. Habituation may eventually result in a zero or asymptotic response but can be instantly recovered if the stimulus changes or a new, stronger stimulus is applied.

Sokolov [13] and Groves and Thompson [15] theorized that habituation occurs via a central process, in which the combined responses of several neurons and interneurons dictate the final response. Sokolov described this system as consisting of afferent, extrapolatory, and comparator neurons. Within the system, comparator neurons compare the afferent signal of the incoming stimuli with the previous response of the extrapolatory signal to determine the organism’s final response [13]. Groves and Thompson’s “Dual-Process Theory” similarly theorized that the combined effects of habituation (reduced response) and sensitization (amplified response) interneurons determine the final efferent outcome [15]. Another model of habituation is Ramaswami’s “Negative Image Model”, which proposes that the brain creates an inhibitory image of repetitive stimuli and uses this to predict incoming stimuli and suppress the signal to higher regions of the brain, thus limiting the response [16]. The convergence of these habituation models is that single neurons or a neural network possess the ability to suppress the input of afferent signals to higher brain centers, thus inhibiting downstream signals and reducing effector responses [13,15–17].

## Cold habituation in non-human organisms

Examples of cold habituation are found across the evolutionary spectrum and point to a conservation of the response. Species as varied as fruit flies, roundworms, rats, and sheep have been shown to exhibit cold habituation. These studies of non-human organisms give insight into the mechanisms that may play a role in human cold habituation.

In *Drosophila melanogaster*, studies have tested the fly’s ability to recover following a 0°C air exposure after growing in temperatures of 12, 14, 17, 21, 25, 28 and 31°C [18]. No differences in the country of origin (Kenya vs. France) were found in the fly’s ability to recover after developing in colder air temperatures, suggesting that phenotypic adaptations to cold exposure in *Drosophila melanogaster* are plastic and more important than genetic variability. Several explanations were given by the authors, one of which is that the adaptation may be a by-product of general functional changes related to growth temperature, such as an increase in the level of unsaturated fatty acids and thus an ability to maintain cell membrane fluidity and normal cell function. However, it is possible that *Drosophila melanogaster* innately possessed the ability to adapt to the cold. Despite evolutionarily being an African native, fruit flies were exposed to low temperatures in the African mountains before migrating to more temperate regions. Thus, although there is the idea that adaptations to cold must have occurred once in Europe, the machinery for this adaptation may have already existed. In some ways, this is similar to the idea that human beings did not adapt to the cold until migrating to colder climates. However, classical experiments demonstrated cold adaptations in Kalahari Bushmen [19], suggesting that, as in the fruit fly, humans who migrated from Africa to other regions may have already possessed the ability to physiologically adapt to cold climates.

Much work has also been done with the nematode, *Caenorhabditis elegans* (*C. elegans*). This roundworm lives in temperate environments (e.g., Scandinavia and the northern United States) and is subject to relatively low temperatures during the year. A number of studies examined the mechanistic underpinnings of cold habituation and tolerance in *C. elegans*. The pathways for cold habituation in *C. elegans* are quite varied and complex with light and pheromone sensing neurons, known as ASJ neurons, implicated in rapid (2–3 hours) cold habituation and tolerance [20]. When *C. elegans* lives at 20 or 25°C and is acutely placed into a 2°C environment, most worms do not survive. However, if the nematode is placed in a 15°C environment for 2–3 hours, exposure to 2°C air does not kill the worm and results in close to a 100% survival rate. Multiple signaling pathways have a role in this reaction, beginning with cold being sensed by ASJ neurons which in turn leads to a cascade of events regulating the response. Cold exposure increases DAF-16/FOXO expression and positively regulates habituation through gene expression of the delta-9 desaturase gene, which is important for cold tolerance in many animals [20]. Other potential regulators of the cold response in *C. elegans* are degenerin/epithelial Na+ channel (DEG-1) mechanoreceptors, endoribonuclease, and potassium channels [21]. Identifying such regulators of cold tolerance in *C. elegans* may be useful in understanding temperature habituation in other animals.

Small mammals also demonstrate physiological adaptations to prolonged cold exposure [22]. When rats are housed in a 5°C environment for 6 weeks, the sensitivity of central and peripheral thermoreceptors that are responsive to low temperatures decreases while the sensitivity of those receptors responsive to warm temperatures increases [22]. These findings are consistent with observations that organisms allow for a greater reduction in core temperature before activating cold defense responses. In cold-adapted cats (5 vs 30°C ambient air), the average dynamic peak frequency of nasal cold fibers during a 5°C cooling perturbation is significantly reduced compared to non-adapted cats [23]; however, this change in thermoreceptor activity has only been observed following long term (~4.5 yrs) cold exposure and not short-term (2 mos.) cold exposure [24,25]. Nonetheless, these studies raise the question as to whether reduced sensory input or thermoreceptor sensitivity contribute to the blunted thermoeffector responses in humans.

Slee et al. studied cold habituation in sheep during three 2-week treatment regimens: continuous exposure to 30°C air, continuous exposure to 8°C, and finally intermittent cold shock, which consisted of 30°C air exposure disrupted by brief −10°C air exposures. During and at the end of these conditioning treatments, the sheep also received two acute cold air exposures at −20°C. They found that the sheep continuously exposed to 8°C exhibited vasoconstriction at a lower skin temperature, perhaps indicative of a peripheral habituation. In contrast, the sheep exposed intermittently to more severe temperatures, had a reduced shivering response, suggestive of a centrally-mediated metabolic habituation [26].

This brief presentation of animal studies gives insight into the various mechanisms that may be involved in cold habituation across different species. The exact mechanisms that mediate these responses, and whether habituation of responses to the cold are an evolutionary mechanism innate in all species, remains to be determined.

## Human physiological responses to acute cold exposure

**Figure 1.**
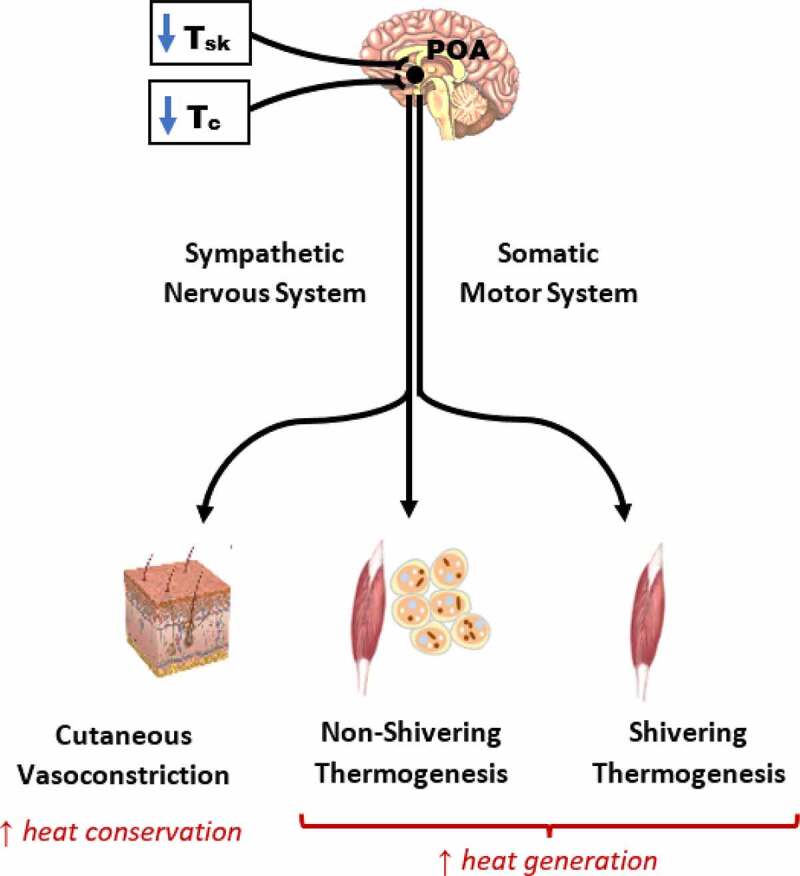
Regulation of physiological thermoeffector responses to cold exposure. Decreases in mean skin temperature and core temperature are sensed by peripheral (skin) and central thermoreceptors. Cutaneous and central afferent signals are integrated in the preoptic area of the hypothalamus, which elicits insulative (heat-conserving) and metabolic (heat-generating) thermoeffector responses. Sympathetic signals descending from the pre-optic area mediate cutaneous vasoconstriction and non-shivering thermogenesis, while descending somatomotor signals activate shivering thermogenesis. POA, preoptic area; T_c_, core temperature; T_sk_, skin temperature.

To provide a foundation from which to characterize cold habituation, this section gives a brief overview of the typical human physiological responses to acute cold exposure. The primary responses for regulating body temperature during acute cold exposure include cutaneous vasoconstriction and increased thermogenesis (Figure 1). The initial physiological response to a cold environment is skin vasoconstriction, which decreases skin blood flow and lowers skin temperature. By reducing convective heat transfer between the body’s core and shell (skin, subcutaneous fat, and skeletal muscle), peripheral vasoconstriction increases thermal insulation and protects against a fall in deep body core temperature. Vasoconstriction occurs when skin temperature decreases below 35°C and is maximal when skin temperature is 31°C or less [27].

Another vasomotor response, cold-induced vasodilation (CIVD), exists in acral skin regions (e.g., palmar aspects of the hands and sole of the foot) and modulates the effects of vasoconstriction [28,29]. Periodic fluctuations of skin temperature follow the initial decline during cold exposure, resulting from transient increases in blood flow to cooled digits. Evidence suggests that CIVD is protective against local cold injuries [30–32], although evidence linking CIVD response to prediction of injury is equivocal [33,34]. The response is modulated by changes in deep body temperature [35–37], but data remain inconclusive on the exact mechanisms that mediate CIVD, as there appears to be evidence for both central [38] and peripheral [39] mediation.

Acute cold exposure also elicits an increase in metabolic heat production. In humans, most cold-induced thermogenesis is attributable to skeletal muscle contractile activity. Humans initiate this thermogenesis either by voluntarily modifying behavior, that is, increasing physical activity (e.g., exercise, increased fidgeting), or by shivering. Shivering, which consists of involuntary repeated rhythmic muscle contractions during which most of the metabolic energy expended is liberated as heat and little external work is performed, may start immediately or after several minutes of cold exposure, and is initiated by a decrease in skin temperature, with a fall in core temperature providing the greatest stimulus. Shivering usually begins in the torso muscles, then spreads to the limbs [40]. The intensity and extent of shivering vary according to the severity of cold stress (e.g., air or water exposure, change in core temperature). Heat production during shivering is about 200 to 250 W during resting exposure in cold air but often exceeds 350 W during resting immersion in cold water [41]. Humans can additionally increase metabolic heat production by non-shivering thermogenesis (NST). A series of papers [42–44] revealed that humans have brown adipose tissue (BAT) that becomes active upon cold exposure. NST also occurs in skeletal muscle. While this review will describe the broader adaptive changes in NST following repeated cold exposure, readers are directed to excellent in-depth reviews by van Marken Lichtenbelt and Schrauwen, Blondin et al., and Carpetier et al. [45–47] for additional information on the metabolic and molecular pathways of NST in cold-exposed humans.

**Figure 2.**
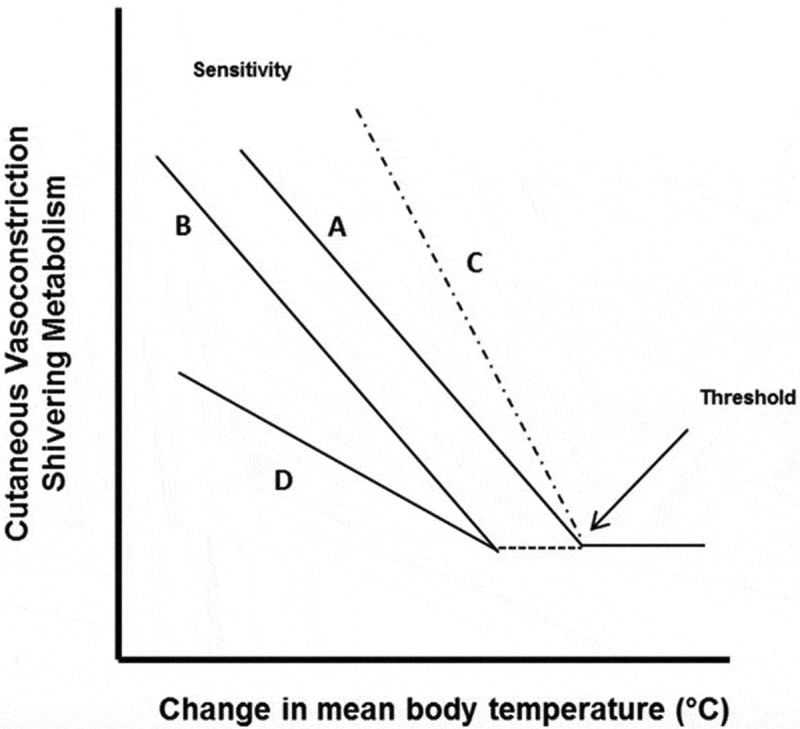
Representation of the thermal effector response (vasoconstriction, shivering) to a change in mean body temperature (ΔMBT) relationship. As mean body temperature decreases a thermal effector response (e.g., shivering) is elicited and increases (line A). The inflection point where this increase occurs is the threshold. The slope of the effector-ΔMBT relationship represents the sensitivity of the response. Line B denotes a response where the threshold is shifted, such that a thermal effector response does not occur until a larger ΔMBT occurs. In Line C, there is no threshold shift, but a change in the sensitivity of the response. For this example, line C denotes a greater sensitivity to a ΔMBT, that is, there is a greater effector for a given ΔMBT. Line D denotes both a threshold and sensitivity change. Reproduced from Castellani and Young, 2016 [2].

Reflex thermoregulatory responses to cold exposure are produced by a series of integrated neural mechanisms. Afferent signals from the skin are sensed in the preoptic area of the anterior hypothalamus, from which efferent signals arise causing cutaneous vasoconstriction and shivering thermogenesis [48]. Cutaneous vasoconstriction and NST are mediated by the sympathetic nervous system and downstream adrenergic and noradrenergic mechanisms, whereas shivering thermogenesis is driven by the somatic motor system (Figure 1). The control of these efferent responses during a reduction in mean body temperature (integration of core and skin temperature) is depicted in Figure 2. The threshold is defined as the temperature point where the effector response is initially activated, whereas the sensitivity of the response is denoted by the slope of the mean body temperature to effector response. A shift in the response threshold is often considered to be the result of a central modulation, whereas a change in the response sensitivity reflects modulation at the peripheral level (i.e., the cutaneous microvasculature) [49–52]. Changes in either the threshold or slope of the vasoconstrictor or shivering responses are a hallmark of adaptive responses to cold. In the context of habituation, higher skin temperatures and reduced shivering thermogenesis are likely due to an *increased* threshold (i.e., delayed onset due to a greater change needed to elicit the response) and/or a *reduced* slope (i.e., lower sensitivity) of the cutaneous vasoconstrictor and shivering effector responses.

Acute cold exposure also causes changes in other physiological systems, including the cardiovascular system. The common response that occurs over different types of cold exposure (whole-body air and water, hand immersion) is an increase in mean arterial pressure (MAP), which is primarily mediated by an increase in total peripheral resistance [53,54]. Furthermore, prior and concurrent to vasoconstrictor and metabolic thermoeffector responses, an immediate cardiorespiratory response occurs during accidental cold water immersion and is known as the cold shock response (CSR). The CSR is characterized by a large gasp for air followed by an acute increase in cardiovascular (HR, MAP) and respiratory (tidal volume, breathing frequency) responses [55,56]. As cold water exposure continues, vasoconstrictor and shivering responses are increasingly engaged to defend body core temperature.

## Habituation in cold air

Habituation of peripheral and metabolic responses to cold occurs most often during repeated moderate cold air exposures. Due to its lower conductivity, cold air cools the body much slower than cold water at the same temperature, therefore creating a milder cooling environment. It is this less severe environment that likely allows for habituation of physiological effector responses, rather than systemic insulative or hypermetabolic adaptations.

### Natural cold air exposure

Cold habituation has been a hallmark response in individuals who live in cold regions of the world. Studies in Inuits and Lapps have demonstrated a blunting of both the metabolic and peripheral responses to cold air exposure, compared to control subjects. Specifically, studies have observed higher hand blood flow [57], finger temperatures [58], and forearm blood flow [59] in Inuits during cold water and cold air exposure, all suggestive of an attenuated vasoconstrictor response to cold in individuals living in cold regions year round, but with access to warm clothing and shelter protecting them against large decreases in core temperature. The Lapps have also been shown to have higher skin temperatures as well as a shift in shivering threshold [60] in lower ambient temperatures, while native Peruvians who experience low temperatures in coastal and highland regions show higher finger temperatures linked to earlier CIVD cycle onset, higher initial cycle onset temperatures, and a greater number of rewarming cycles [61].

Multiple studies have also been completed on indigenous people who live in relatively temperate environments but experience low temperatures at night due to insufficient clothing or shelter to protect them. Experiments conducted on indigenous Australians, for example, showed a shivering habituation [62–64], compared to control subjects. Likewise, indigenous South Africans showed a similar habituation of the metabolic response [1,65]. Table 1 contains a summary of studies on cold habituation in indigenous populations.

**Table 1.** Cold habituation in indigenous populations.

	Study Sample	Habituation Length	Habituation Temperature	Cold Testing Procedure	Results/Findings
Brown [57]	22 M Eskimos	Lifetime	Seasonal Variation; outdoor temp 0–19°C, indoor temp 20°C at time of collection	Room air at 20°C, Hand/Forearm Immersion in 5–45°C water-bath	↑ hand blood flow (11–313%), ↓rate of reduction of hand blood flow in Eskimo vs. white control at water-bath temperatures between 5–42.5°C
Brown [59]	29 M Eskimos	Lifetime	Seasonal Variation; indoor temp 20°C (RH 50–60%) at time of collection	Room air at 20°C (bared and clothed forearm), Hand/Forearm Immersion in 5–45°C water-bath	↑ clothed forearm blood flow at any given water bath temperature <45°C vs. white control; ↑ forearm blood flow and ↓ muscle temperature in Eskimo during the 2^nd^ hr of 10–38°C water bath; with 5°C ↓ muscle temperature was faster and larger
Scholander [63]	6 M Australian Aborigines	Lifetime (clothed during the day and nude overnight)	Air temperature in the region typically drops as low as 0°C in the early mornings	Natural night time exposure and observation (naked with fire and naked without fire)	In light sleeping bags without fire, ↓T_sk_ at the foot to 12–15°C in natives; natives slept soundly with unchanged resting heat production; white controls ↓ T_sk_ similarly but ↑ shivering and metabolism
Hammel [64]	8 M Central Aborigines; 7 M Control Whites; 9 M Tropical Aborigines	Lifetime (Aborigines clothed during the day and nude overnight)	Air temperatures at night dropped to 0–5°C	Natural night time exposure and observation in the winter, simulated cold night exposure in the summer using refrigerated meat van	Metabolism of central natives ↓ continually throughout the night with a Q10 of ~2 in summer as in winter, body temperature ↓ at a greater rate vs. whites; the metabolic rate adjustments to cold of the average tropical native fell between that of the control whites and the central natives
Andersen [60]	14 M Lapps	Lifetime	Seasonal Variation	Night time exposure to 0°C; in wind proof blanket sleeping bag on wire mesh sheet with extra woolen blankets and reindeer skins, last 2 layers removed after first 2 hrs	↑ shivering threshold compared to controls, ↓ metabolic heat production, ↓ T_c_, ↑T_sk_
Miller [58]	8 M, 3 F, 8 boy, 4 girl Eskimos	Lifetime	Seasonal Variation; between −20–5°C at time of collection	Cold air exposure (−6.6 to −7.2°C for men for 45 min, −2.6 to −4.8°C for women for 35 min and children for 30 min); wore parkas to ensure thermal comfort but no gloves	Adult male Eskimos ↑ hand and finger temperatures compared to white men; Eskimo children maintained finger temperatures at a nearly identical level as unacclimated white men despite ↓ hand volume; ↓ cold-induced pain with Eskimos; White males exposed to the cold regularly exhibited hand and finger temperature nearer to that of the Eskimos with considerable individual variability
Little [61]	41 Nunoa, Peru natives; 10 Mollendo, Peru natives; 8 whites	Lifetime	Nunoa and Mollendo natives share similar culture and ancestry but Mollendo is warmer throughout the year (10 to 19°C vs. −5 to 8°C)	Local exposure of the hand or foot to 0°C air for 60 minutes in the morning and afternoon; During foot cooling, Peruvians were tested at altitude (4000 m) while the whites were tested at ~ sea level	More rapid ↓ in toe and foot T_sk_ among white subjects during the first 30 min of foot cold exposure; with hand cooling, finger T_sk_ showed a steeper ↓ amongst whites that began to level off at the 40^th^ minute of cold exposure; Peruvians showed CIVD cycling at much ↑ temperatures than whites but with low amplitude during foot cold air exposure; ↔ between Nunoa and Mollendo Peruvians

Short-term studies of repeated or continuous cold air exposure within subjects have similarly resulted in habituation of heat-conserving thermoeffector responses. Bruck et al. exposed a group of minimally-clothed students to air temperatures that varied between −5 and 5°C, based on individual resilience, for 1 hour on 4–7 occasions over 14 days [66]. In the same study, the investigators observed soldiers who trained and slept in the cold for 10 days in temperatures varying between −2 and 12°C. Of the student volunteers, 54% experienced a hypothermic habituation, i.e., a lower core temperature, reduced cold sensation, and blunted metabolic response with a delay in shivering, 23% showed only a metabolic habituation, and 23% showed no changes [66]. Of the soldiers, 44% showed habituated metabolic and cold sensation responses. The habituated metabolic response was more frequently observed in the student group than in the soldiers. The authors hypothesized that the soldiers showing no changes may have already been cold acclimatized prior to the experiment, as their baseline shivering thresholds were similar to the post-training shivering thresholds of the adapted soldiers. Other group differences may be attributed to differences in training load during the experiment. Clothing may have also played a role as the soldiers were dressed in a field uniform during the 10-day training exercise, whereas the students wore a bathing suit during the resting repeated exposures, potentially resulting in a more severe cold stress.

A study by Muller et al. compared the responses of cold weather athletes with those of less-acclimatized individuals during a standardized cold air exposure. Through sport, the athletes were exposed to ambient temperatures between −8 and 7°C for about 2h/day in the winter months of January-March, during which they trained for about 30 min out of each 2 hour exposure. The non-athlete student population trained a similar amount of time per week, but were only exposed to low temperatures during necessary outdoor activities such as walking to class. Consistent with a habituation-type adaptation, the athletes had reduced cold pain, a blunted reduction in hand temperature, and an attenuated metabolic response during a controlled exposure to 5°C air for 90 min, showing that the 2h exposures to winter temperatures resulted in a habituated response compared to the group of students who were less exposed [67]. These observational studies give evidence that habituation is a naturally occurring adaptation to those who spend time outdoors in low temperatures. A summary of longitudinal natural cold air exposures can be found in Table 2.

**Table 2.** Longitudinal natural cold air exposure.

Reference	Study Sample	Habituation Length	Habituation Temperature	Cold Testing Procedure	Results/Findings
Carlson [139]	7 M	16–18 hr daily exposure for 14 days	Outside air temperature varied from −5 to −17°C; wore adequate clothing to prevent discomfort	Passive observation to natural exposure	Slower initial rate of hand T_sk_ ↓ with hand T_sk_ maintained at a greater temperature after 14 days of exposure; CIVD amplitude ↓ over the 14 days
Bruck [66]	9 M (Study B)	10 day, 24 hr exposure	−2 to 14°C	Pre and Post cold test: 30 min at 28°C, linear decrease of 0.5°C per minute to 5 to −5°C for 1 hr or until shivering	Habituation temperature deemed a ‘mild’ cold stress; only 4/9 subjects saw cold habituation: shivering threshold occurred at ↓T_b_, ↑ resting metabolic rate, cold sensations occurred at ↓ T_b_, and T_es_ ↓ following the 10 day exercise
Bodey [140]	7 M Caucasians	1 continuous year in Antarctica	Outdoors: ~0 to −20°C with mean wind speed as high as 10 m/s for 2–3 hrs per day; Indoors: 21–23°C with brief but frequent lightly clad pulses of ambient cold	Standard cold stress test of 10°C for 2 hrs + rewarming completed before (in Melbourne) and 4 times during the year in Antarctica	Within a month of arriving in Antarctica, ↓ peripheral temperatures and ↓ T_re_ cooling rate in the second hour of cold stress; After 9 months, ↑ T_sk_ and ↓ T_re_ in the second hour of cold stress along with an ↑ in peripheral rewarming rate; ↓ in plasma cortisol, ↑adrenaline excretion established in late standard cold stresses; ↑ delay in the onset of shivering, ↓ T_sk_ at shivering onset, and ↓ NE observed in late standard cold stress
Bittel [141]	1 M French doctor	63 day journey from arctic Canada to the North Pole (1100 total km)	Ambient temperatures varied between −52 and −12°C; wore light-weight insulated clothing, skied pulling a 50 kg sled daily	Pre and post cold test: 2 hr exposure to 1°C (wind speed 0.8 m/s, RH 40%) laying on a wire mesh bed wearing only swimming trunks	After his journey, a general hypothermic-hypometabolic adaptation characterized by a ↓ T_rec_ and metabolic heat production and an ↑ skin temperature of the extremities; authors state that tympanic temperature, as representative of the CNS temperature, ↑ despite T_rec_ ↓ suggests a redistribution of blood volume to the CNS
Livingstone [117]	4 M Canadians	91 day North Pole ski expedition	Unknown	Passive 10°C air exposure for 90 min + Finger 0°C 30 min ice water bath immersion	10°C air: After 91 days of exposure, ↑ time to onset of shivering, smaller ↑ in metabolism in response to cold; 0°C water bath: ↑ CIVD response
Muller [67]	14 M; 6 cold-acclimated American football players	2 years	2 hrs/day at 0°C (range −8 to 7°C) from January-March	90 min resting at 5°C, 30 min exercising at 50% VO2peak at 5°C, 60 min post-exercise recovery in 5°C	At 5°C rest, cold-acclimated football players had ↑ finger temperatures, ↓ metabolic rate, ↓ hand pain, ↓ negative mood, ↔ dexterity; With continuous exercise in 5°C, cold-acclimated football players had ↑ finger rewarming after 20 min that occurred at ↓ T_c_

### Laboratory cold air exposure

Multiple studies have exposed individuals to low temperatures in a controlled environmental chamber to more closely examine the ability of humans to adapt to cold environments over days, weeks, or months (Table 3). Individuals exposed to cold air (~12-13°C) for 8 h/day for 31 days, for example, demonstrated significant reductions in shivering and lower core temperatures in the cold [68]. Interestingly, seasonally acclimatized individuals who were tested in March did not show a reduction in total metabolic heat production, suggesting a compensatory increase in NST. The minimally acclimatized individuals who were tested in September or October, on the other hand, had a higher baseline heat production in the cold and demonstrated a progressive reduction in total heat production to a level that, after the 31 days, matched the seasonally acclimatized individuals. Kreider et al. continuously exposed a group of soldiers to 15°C for 14 days wearing only shorts and observed higher toe temperatures and lower core temperature at night when soldiers were covered with a sheet and blanket. These results are indicative of a peripheral adaptation in the extremities, though the authors also recognized that the higher toe temperature may have reflected a greater post-ischemic reactive hyperemia [69].

**Table 3.** Cold air laboratory studies.

Reference	Study Sample	Habituation Length	Habituation Temperature	Cold Testing Procedure	Results/Findings
Kreider [69]	5 M Soldiers	Continuous 14 days	15.6°C (RH 40–50%, wind <1mph); wearing only shorts, with sheet and blanket at night	Longitudinal passive observation	Nocturnal T_rec_ ↓ on the later cold days: Nocturnal toe temperatures were 15°C ↑ on the later cold days
Davis [68]	10 M maximally-acclimatized; 6 M minimally-acclimatized	8hrs daily for 31 days	11.8°C, wearing only shorts	Longitudinal passive observation; measurements made with subject nude during a 2 hr length of cold room exposure	By the 14^th^ day, shivering in both groups ↓; Metabolic heat production ↔ in maximally-acclimated group but ↓ in the minimally-acclimated; in both groups, T_rec_ ↓ after the 31 day exposure; Extremity temperatures ↔ in maximally-acclimated and slightly ↓ in the minimally-acclimated group
Keatinge [142]	14 M: 5 M passive cold exposure, 5 M warm room exposure, 4 M performed physical work in warm room	7.5 hrs daily for 19 days	6°C, air movement of 30 cm/s, wearing shorts, socks, and boots	Measurements taken the first and last day with cold exposure (6°C)	Physical activity group had ↓ early metabolic response to cold and maintained ↑ forearm T_sk_ after physical training; Passive cold exposure group had ↑ in the early daily metabolic response with a ↓ in metabolic rate at the end of each day, ↑ rate of T_rec_ decline across daily exposure, ↔ T_sk_, T_rec_, or intramuscular temperature; BMR unchanged in either group
Bruck [66]	14 M	1 hr exposure 4–7x within 2 weeks	Ambient temperature decreased from 28°C to 5°C to −5°C, wearing a bathing suit in a resting position	Longitudinal passive observation throughout repeated exposures	In 2/3 of subjects, metabolic heat production and shivering threshold occurred at ↓ T_b_ and T_es_ following repeated exposure, ↓ thermal discomfort and cold sensation, ↔ basal metabolic rate or T_sk_
Mathew [120]	15 M soldiers	4 hr exposure to cold air daily for 21 days	10°C, wearing only shorts	Standard cold test (10°C air for 2 hrs) at days 1, 6, 11, 16, and 21	By day 21, ↑ RMR, smaller ↓ in T_b,_ ↓ shivering, ↑ CIVD and thermoregulatory efficiency, less rise in BP and HR during cold pressor response
Silami-Garcia [71]	10 F; 5 F cold-exposed, 5 F CON	Cold exposed: 10x for 1 hr within 2 weeks; CON: 2x within 18 days (for response measurement)	10°C air, clothing unknown	Measurements taken the first and last exposures (10°C air)	After ~5 exposures, cold-exposed women ↑ time to onset of shivering and ↓ metabolic heat production, ↔ T_sk_, T_b_, T_rec_, or big toe temperature
Armstrong [143]	4 F	10 days of daily cold air exposure	22°C for 45 min + 4°C for 45 min; wearing t-shirt, shorts, and cotton socks	Longitudinal passive observation: RMR measurements taken daily throughout exposures	During cold air exposure, RMR peaked at 31% VO2peak by the 5^th^ min in Day 1, peak RMR on day 5 was ↓ (24%) and persisted through days 8 and 12; steady state RMR followed a similar ↓ trend beginning at day 5
Hesslink [8]	16 M; 8 M triiodothyronine supplementation, 8 M placebo	80 total (10x/week) 30 min exposures	4.4°C air, wearing shirt, shorts, socks	SCAT in basal conditions in January and again in March	↔ BMR, T_re_; metabolic heat production, mean arterial pressure, and norepinephrine ↓ for all subjects in March, maintenance of T_4_ and TSH is not essential for habituation
Leppaluoto [107]	6 M	2 h daily for 11 days	10°C air, air velocity <0.2 m/s, humidity of 204 g/m^3^, only wearing shorts	Longitudinal passive observation: measurements taken daily for 11 days	↓ general cold sensations and those of hand and foot after the first exposure that remained throughout; ↑ T_sk_ and ↓ SBP after 4–6 exposures that disappeared by experiment end; forearm T_sk_ specifically ↑ after 4–6 exposures and remained to some extent throughout, ↓ NE response on days 5 and 10, ↔ T_re_
Makinen [7]	10 M	2 h daily for 10 successive days	10°C air, air velocity <0.2 m/s, 50% RH (vs. 25°C air control), wearing shorts, socks, athletic shoes	Longitudinal passive observation: measurements taken days 1 and 10	With repeated 10°C exposure, ↑ T_sk_ and ↓ NE (24%); ↑ high frequency HRV power; ↓ increase in HR and blood pressure (specifically ↓ DBP) during handgrip testing
Park [144]	8 M	2 h of morning cold exposure + 2 h afternoon running/rest in the heat for 14 consecutive days	Cold: 10°C 40% RH with 0.31 clo Heat: 30° 60% RH with 0.28 clo	Pre- and Post-exposure program: cold tolerance test (10°C, 40%RH) with 0.21 clo – 60 min passive chair sitting in the cold	↓ SBP, DBP, MAP post exposure; ↔ T_sk_ or metabolic heat production; ↓ in thermal sensation but ↔ thermal comfort

In two studies investigating adaptations to short, intermittent cold air exposures, one by Leppaluoto et al. and the other by Makinen et al., participants were exposed to 10°C air for 2 h/day for 10–11 days. Both studies reported attenuated vasoconstrictor and BP responses, as well as a decrease in cold thermal sensation [6,7]. Makinen [70] also demonstrated a decreased metabolic response, which agrees with findings by Silami-Garcia and Haymes [71] who demonstrated an increase in the time to onset of shivering and a decrease in heat production in women exposed to 10°C air for 1 h/day for 10 days. A longer intervention by Hesslink et al. exposed participants to 4.4°C air for 30 minutes, twice a day for 8 weeks and, although a more severe air temperature, the short exposure time resulted in a similar cold habituation, including reduced BP and metabolic responses with a delay in shivering thermogenesis [8].

Collectively, these studies show that the habituation of metabolic, vasoconstrictor, and sensation responses to cold air exposure can occur following not only prolonged exposure, but also repeated exposures that are short in duration (8h or less) and under moderate cold conditions (0–12°C).

## Habituation in cold water

Adaptations to cold water exposure vary across different occupational and laboratory settings and often demonstrate more variable types of adaptation based on surface area exposed and duration of exposure.

### Local cold water exposure

Habituation can be produced even if cold water exposure is limited to relatively small regions of the body. For example, fishermen and fish filleters work long hours every day with one or both hands immersed in cold water, and have been shown to maintain higher finger and hand temperatures and lower systemic BP during hand immersion in cold water compared to control subjects [72–74]. Slaughterhouse workers who handle cold meat tend to show similar adaptations [75], although the adaptions are not as pronounced as the Gaspé fisherman, likely due to a weaker stimulus. This suggests that repeated cold exposure of the extremities can produce localized habituation of vasoconstrictor responses. Another interpretation of these warmer skin temperatures, suggested by Nelms and Soper [74], is an adaptive enhancement of the CIVD response, though more recent studies examining the short-term adaptability of the CIVD response are equivocal [29,76]. It is important to consider that selection may play a role in occupationally based studies such that those with enhanced physiological mechanisms for coping with the cold may have chosen this type of occupation due to their increased ability to handle cold with less decrement in comfort and function, rather than the repeated cold exposures leading to habituated responses [29]. A summary of the responses to occupational cold water exposure of the hands is presented in Table 4.

**Table 4.** Occupational hand cold water exposure.

Reference	Study Sample	Habituation Length	Habituation Temperature	Cold Testing Procedure	Results/Findings
LeBlanc [72]	14 Gaspe Fishermen	Occupational (intermittent but daily), several hours per day for 2–25 yrs	Sea water temperature: 11°C, Air temperature: 9.4–12.4°C	10 min 30°C hand water bath, 10 min 2.5°C water hand immersion, 10 min in room air	With hand cold bath, Gaspe fishermen had ↓ blood pressure, ↑ finger temperature, ↓ reported pain, and ↑ heat flow from the hands; ↑ number of mast cells present in the hand skin of the fishermen
LeBlanc [145]	Exp #1: 10 Gaspe Fishermen; 11 CON; Exp #2: 6 Gaspe; 7 CON	Occupational (intermittent but daily), several hours per day for 2–25 yrs	Sea water temperature: 11°C, Air temperature: 9.4–12.4°C	Exp #1: 5 min 2.5°C foot immersion; Exp #2: 5 min 2.5°C hand immersion 4 months after end of fishing season	Exp #1: Gaspe fishermen had ↓ BP response, ↓ foot T_sk_; Exp #2: Gaspe fishermen able to retain ↓ BP response 4 months outside of seasonal exposure, both Gaspe and CON had ↓ BP response in winter vs summer
Nelms [74]	11 British fish filleters; 9 CON	Occupational: left hand water immersion or cold fish handling 4–8 hrs/day	−1 to 8°C water exposure; concurrent general cold wind exposure dockside	0°C ice water hand immersion	Earlier onset and greater magnitude of vasodilation in the filleters, T_sk_ ↑ with immersion during initial vasoconstriction and subsequent vasodilation, ↓ acute and lasting pain sensations
LeBlanc [146]	7 Gaspe Fishermen; 7 CON	Occupational (intermittent but daily), several hours per day for 2–25 yrs; data collected 1 ½ months into fishing season	Sea water temperature: 11°C, Air temperature: 9.4–12.4°C	Naked 1 hr 15°C cold air exposure	Gaspe had ↑ T_sk_, ↑ shivering (especially with those with greatest T_sk_) but ↔ metabolic heat production; ↓ in cold pain
Enander [75]	10 M occupationally cold exposed; 10 office workers	Daily work exposure to cold air	5–10°C air	Two cold water hand immersion tests (immersion of rubber-gloved hands to the wrists in 10°C water for 2 min), one in 10°C and one in 20°C ambient air, recovery from hand immersion for 30 min in respective ambient air temp	Those not cold exposed rated ↑ cold sensation and frequency of pain ratings from cold water immersion, especially in ambient 10°C

Habituated responses to repeated local cold exposures have also been shown in the laboratory (Table 5). Leftheriotis et al. immersed the hand and forearm in 5°C water for 20 min on 30 consecutive days. The cold-adapted group showed reduced cold sensation and higher skin temperatures following 5 min of 5°C immersion [77]. Eagen explored local vascular adaptations to cold in a controlled laboratory environment using 125 consecutive days of ice water immersion (0°C) of the middle finger for 10 minutes, six times per day [78]. Using the contralateral middle finger as a control, no difference in finger temperatures were present during immersion between the habituated and control fingers following 125 days of repeated exposure, although cold pain was markedly reduced in the habituated finger. Interestingly, comparison of the immersion response of this contralateral control finger to the finger of completely non-habituated controls indicated an elevated finger temperature, suggesting the 125 day cold immersion protocol was sufficient to reduce vasoconstrictor outflow to both the finger immersed in ice water as well as the contralateral finger.

**Table 5.** Local cold water immersion laboratory studies.

Reference	Study Sample	Habituation Length	Habituation Temperature	Cold Testing Procedure	Results/Findings
Eagen [78]	6 M airmen	125 consecutive days, 10 min middle finger immersion 6x per day	0°C	Identical immersion post testing on day 126,127, and 128	Post habituation, finger temperature of the control contralateral finger during immersion was similar to habituated finger but maximum pain was ↓ for the habituated finger vs control; habituated finger temperature of immersed group was ↑ than that of a separate control group
LeBlanc [81]	16 M	Two groups 8 M each: (1) L hand cold water exposure 2x/day for 5 consecutive days for 4 weeks; (2) cold water immersion of L hand + mental arithmetic	Group (1): 4°C Group (2): 4°C	Pre and post testing consisting of: Test I: immersion of L hand in 4°C water for 2.5 min Test II: Mental arithmetic test Test III: cold water immersion of L hand + mental arithmetic Test IV (only added post): immersion of R hand in 4°C water for 2.5 min	Those in group 2 (cold water immersion + mental arithmetic), did not adapt to the cold water test alone (↔ in blood pressure), only the combination of cold water immersion + mental arithmetic (↓ blood pressure) following habituation; ↓ BP and pain estimation response to cold in one hand did not confer the same adaptation to the opposite hand, rather it appeared to sensitize the response
Zbrozyna [82]	7 M, 4 F	Cold water immersion of one foot for 60 sec 7x/day for 6 days; rewarming bath between intervals	4°C water; 36°C rewarming bath	Longitudinal passive (time-focused) observation	After repeated cold water immersions, ↓ in reactive muscle vasodilation (noticeable even after a single session of immersion) and BP (most significant around immersion day 4) to same stimulus
Leftheriotis [77]	10 M Caucasians; 5 M locally cold-acclimated, 5 M were non-acclimated	Daily immersion of the R hand and forearm in a stirred water bath for 20 min for 30 days	5°C water	Pre and post testing consisting of three tests performed in both 25°C air and after 5 min hand and forearm cooling in 5°C: (1) peak blood flow following ischemia, (2) peak blood flow following exercise, (3) peak blood flow following ischemia and exercise combined	After repeated cold exposure, lesser ↓ in skin temperature; peak blood flow following ischemia and ischemia+exercise in the finger and forearm was ↓ in the cooled condition only in those who were cold-acclimated; forearm peak blood flow following exercise was ↓ in the cooled condition only in the cold-acclimated males indicating muscle blood flow was also ↓; overall, cold-acclimated males showed ↓ vasodilatory responses only when exposed to cold
Carman [83]	38 M & F	9 days of cryokinetic treatments (5 cold immersions interspersed with 3-min of exercise) to R ankle + 1 day to L ankle; cold water immersion included 1 20-min immersion followed by 4, 5-min immersions	1° or 5°C water; wearing toe caps	Longitudinal passive (time-focused) observation; Days 9 & 10 R ankle treated with opposite temperature and L ankle was treated with habituation temperature	From combined 1 and 5°C data: Sharp ↓ in cold pain from days 1–5, but no difference from 5–8 days; instep was the most frequent location of pain for the first 3 days and the choice of “no specific location” ↑ steadily from day 2–8; on days 9 & 10 pain in the L limb was ↑ than that at the end of the R limb habituation but ↔ to day 1 of habituation indicating non-adaptation transference; R limb immersion in a lower temperature resulted in ↑ pain than that perceived on day 8 indicating temperature adaptation specificity
Savourey [80]	8 M (euthyroid)	Standing ice water immersion of lower limbs up to 20 cm above the knees 2x/day, 5 days/wk for 1 month; duration of immersion was to tolerance (~5 min at the start and ~60 min by the end)	0–5°C	Pre and post testing using Standard Cold Air Test (SCAT): 1°C air exposure for 2 hrs, nude, at rest	After acclimation, slightly ↓ TT_3_ both before (−18%) and after (−11.7%) correction for change in plasma volume, ↓ T_c_ suggests a hypothermic general cold adaptation
Savourey [79]	8 M (euthyroid)	Ice water immersion of lower limbs up to 20 cm above the knees 2x/day, 5 days/wk for 1 month; duration of immersion was to tolerance (~5 min at the start and ~60 min by the end); 40 total immersions	0-5°C; wearing bathing suit, shirt and waistcoat to prevent shivering	Pre and post testing using cold foot test (CFT; 5°C water immersion of R foot up to the knee for 5 min) + Standard Cold Air Test (SCAT; 1°C air exposure for 2 hrs, nude, at rest)	↑ T_sk_ of lower limbs and ↓ related pain during CFT and ↓ T_rec_ and mean T_sk_; ↔ metabolic heat production or lower limb skin temperatures during SCAT; ↓ plasma NE over the course of habituation but ↑ in NE during SCAT after habituation; post cold acclimation: ↑ FT_3_ and slight ↑ TT_3_ from pre-control vs 40^th^ immersion but ↔ TT_4_, FT_4_, and TSH (termed “T_3_ polar syndrome”)
Tipton [99]	13 M; 8 habituation, 4 CON	6 3-min head-out immersions over 3 days (2x/day)	15°C; wearing swimming trunks	Pre and post testing: 3-min head-out 10°C cold water immersion wearing swimming trunks	After habituation exposure at 15°C, respiratory rate, inspiratory minute volume, and HR ↓ over the first 30 sec (as well as the rest of the 3-min immersion) of exposure in both 15 and 10°C water; habituation can be achieved with warmer water than that for which adaptation is required
Kolev [147]	5 M, 5 F	10 cold water immersions of one foot for 30 sec with inter-stimulus intervals ranging from 3.5–5 min, rewarmed during withdrawal intervals vs. internal caloric stimulation	5°C for cold water immersion, 37°C rewarm	Longitudinal passive (time-focused) observation	With repeat external cold water foot immersion, ↓ in the red cell flux in the index finger indicating a habituation of the cold microcirculatory reflex (significant ↓ after 7^th^ stimulation); ↔ in T_sk_ of the index finger following the 10 immersions
Geurts [148]	7 M, 4 F Caucasian	Left hand cold water immersion for 30 min, 5d/wk for 2 wks	8°C water	Pre and post testing: neuromuscular function, blood markers, thermal sensation, and temperature responses of both L and R hands assessed in both thermoneutral (~24°C) and cold (8°C) conditions	From pre to post in R vs L hand ↔ in minimum index finger temperature, T_c_, HR, NE, E, NO Endothelin-1, or hand temperature; thermal comfort after 30 min of cold water immersion ↑ in the hand repeatedly exposed to cold, but not in the non-exposed hand
Daanen (76)	9 M, 7 F	Right hand and foot simultaneous immersion 30 min daily for 15 consecutive days	8°C water	Longitudinal observation daily of pain, tactile sensitivity, and skin temperatures of right (trained) hand and foot; pre and post training immersion testing of both right and left (untrained) hands and feet	From first to last immersion, mean toe temperature of the trained foot ↑, but mean finger temperature and number of CIVD reactions ↓ (~30%) in trained hand; no significant differences seen in the untrained limbs; pain ↓ as a function of time and tactile sensitivity ↓ alongside skin temperature; this combination of adaptation may lead to an increased risk of finger cold injuries
Simpson [84]	9 M, 8 F	Single hand cold water immersion to tolerance to a maximum immersion duration of 180 s at baseline and every 5^th^ and 7^th^ day for 3 weeks (total of 7 immersions) in control vs. sleep restricted groups	2–3°C water	Longitudinal passive (time-focused) observation	In the control sleep group, cold pain tolerance time ↑ by 24 s from baseline to week 3, while in the sleep restricted group cold pain tolerance ↑ by 9.5 s

In the lower limbs, Savourey et al. examined adaptations to 1 month of twice-daily partial leg immersions (i.e., up to the thigh) in ice water (0–5°C) that lasted until the participants reached their pain threshold [79,80]. After the month of repeated exposures, participants completed a standardized 5°C foot immersion, during which habituated responses were observed, including higher skin temperatures and a smaller rise in BP. Collectively, these studies point to the existence of vascular adaptations in the extremities following repeated local cold water immersion, though the enhanced vasodilatory and/or reduced vasoconstrictor pathways that contribute to this response remain unclear.

Another subset of cold studies have been utilized to study the ability to habituate to pain. These studies typically involve a severe cold exposure of 1–5°C of a peripheral body part (finger, hand, foot) [81–84] and show that exposing individuals to multiple severe, short-duration cold-water exposures can result in reduced pain sensation and an increased pain threshold. Similar results were observed by Smith et al. when using a thermode to stimulate cold pain [85,86]. Participants allowed the thermode to become on average 1.7°C colder before reporting pain after only 5 bouts. Though we have highlighted just a subset of studies examining perceptual adaptations to cold, this improvement in pain sensation appears to be a consistent adaptation following repeated cold exposure of the extremities.

### Occupational whole-body cold water exposure

The physiological responses of the pearl divers of Korea (Haenyeo) and Japan (Ama) are the best example of adaptations to the chronic declines in core and skin temperature experienced during occupational cold water exposure. These women dive year-round, with average water exposures ranging from 40 min in 28°C in the summer, to 15 min in 10°C in the winter, repeated 2–3 times over the course of a day. Classical divers had very little protection from the cold water, wearing only a cotton bathing suit and therefore had no external insulation from the environment. Due to this lack of external protection, the Ama have been reported to have several adaptations to help protect them from the cold. In the winter, the divers show a 30% higher basal metabolic rate when tested in thermoneutral conditions, and show a suppressed shivering response when immersed in cold water [87–89]. The divers have been reported to have increased tissue insulation, yet are able to maintain a higher blood flow in the lower arms and hands with less heat loss while fully immersed. The authors suggest this is due to “a more efficient countercurrent heat exchange system in the limbs” through which blood may be precooled before reaching the periphery. When only the hand is immersed, the Ama demonstrate a lower hand skin blood flow, yet show a slower reduction in muscle temperature in the lower arm compared to non-divers, thus giving more evidence to a redirection of skin and muscle blood flow [88].

From these reports, we can see that long-term systemic metabolic adaptations occur alongside more complex peripheral vasomotor adaptions. These divers are an especially unique population, as full-body, severe, repeated cold exposure is a rare occurrence in modern society. This population was first studied in the early 1960’s and, five decades later, data from Ama and Haenyeo volunteers are still being published. Recent work has examined the de-acclimatization of older Haenyeo divers following adoption of the wet suit in the 1980’s. Perhaps not surprisingly, data in these older female divers suggest they no longer have the same general systemic thermoregulatory adaptations that were observed in the cotton suit wearing divers [90]. However, the wet suit wearing Korean divers may have developed a habituated, local vascular response, as they show greater minimum and recovery finger temperatures during a standardized cold water finger immersion [91]. Further examples of occupational cold water immersion can be found in Table 6.

**Table 6.** Natural/occupational whole-body cold water immersion.

Reference	Study Sample	Habituation Length	Habituation Temperature	Cold Testing Procedure	Results/Findings
Skreslet [149]	3 M nonprofessional scuba divers	Daily water immersion for 45 days, length of individual dive unknown	Minimum sea temperature of 2.5–3.5°C	Normal sea dives + standard dives in cooled bath to replicate sea temperatures; wearing neoprene suit, gloves, boots	Pattern of acclimatization: 1) unacclimatized: cold stress not met with an ↑ metabolic rate to compensate heat loss, 2) intermediate: ↓ in T_C_ as heat loss is not fully compensated for by metabolism, 3) acclimatized: ↔ T_C_ maintained with minor metabolic heat production
Paik [150]	8 F Korean Ama; 8 F CON	Occupational exposure: 15 min to 2 ½ hrs of full-body exposure year round	10–27°C, season dependent, in cotton bathing suit	6°C hand immersion for 30–60 min	Across seasons, Ama maintained ↑ muscle temperature compared to CON; Finger skin temperature and blood flow ↓; ↑ fraction of venous return via superficial veins; Ama did not appear to undergo CIVD fluctuations; Overall, ↑ vasomotor tone
Dressendorfer [151]	12 M athletes; 6 long-distance runners, 6 long-distance swimmers	~1.5 years (runners averaged ~110 km of road running per week in year-round training; swimmers swam 10 km per week year-round)	Runners: air temperatures of 21–29°C; Swimmers: open water temperatures of 23–25°C	Cold tolerance test: 2 hr head-out circulating (6.4 m/min) water immersion wearing a swim suit in 30°C	Hypothermic insulative adaption in runners that may be related to a vascular mechanism; during the first 75 min of the CT test, T_rec_ in the runners fell 0.3°C/h faster than in the swimmers despite ↔ in metabolic response, calculated insulation values in the runners were ~10% ↑ than the swimmers attributable to elevated nonfat insulation (at a similar level to Korean Ama); marathon training may provide cross-adaptation to cold
Huttunen [152]	6 M, 1 F Russian Long-distance swimmers	Unknown previous practice exposure; 4 days standard exposure, 2x/day	10–14°C	Longitudinal passive observation	↓ rise in diastolic blood pressure on 4^th^ compared to 1^st^ day; Self-determined swimming time lengthened by ~10 min from day 1 to day 4

### Laboratory whole-body cold water immersion

Several controlled laboratory studies have used repeated water immersion over the course of at least 4 weeks to elicit adaptations (Table 7). A study by Lapp and Gee explored adaptations to immersion by reducing the water temperature from 30 to 21.1°C over the course of 8 weeks [92]. Subjects were fully immersed (including the head, with scuba gear) twice per week for 1 h, with a reduction in water temperature each week. Results indicated that by the later immersions, subjects experienced less frequent shivering even though the water temperature was lower than the beginning weeks, suggesting habituation of the shivering response.

**Table 7.** Whole-body cold water immersion laboratory studies.

Reference	Study Sample	Habituation Length	Habituation Temperature	Cold Testing Procedure	Results/Findings
Lapp [92]	3 M, 5 F students	Cold water immersion 2x/week for 1 hr over 8 weeks	Reduced from 30°C to 21.1°C over 8 weeks	Longitudinal passive observation	↑ VO_2_ yet less frequently reported shivering in later weeks despite exposure to ↓ water temperatures
Radomski [93]	11 M; 3 M preadapted (PA) with immersion, 8 M non-preadapted (NPA)	9 daily immersions (20–60 min depending on tolerance) in cold water 20 days before Arctic exposure vs no immersion CON + 16 days in the Arctic	15°C immersion + Arctic (mean temperature −26.8°C)	Nude cold tolerance tests (10°C air for 1 hr resting supine) pre and post Arctic exposure	NPA: ↑ metabolism and T_re_ post Arctic exposure; ↑ urine volume (86%), urinary NE (48%), epinephrine (84%), and 17-hydroxycorticosteriods (34%) PA: ↔ metabolism and ↓ T_re_; ↑ epinephrine (65%)
Young [96]	7 M	Daily 90 min cold water immersion 5 times/week for 5 consecutive weeks	18°C, wearing only swim trunks	Cold air stress test (CAST) pre and post acclimation program: 30 min rest at 24°C, 30% RH followed by 90 min in cold 5°C 30% RH air wearing only swim trunks	Post acclimation: ↓metabolism at 10 min of CAST but ↔ by 30 min, as such shivering onset delayed; T_rec_ ↓ before and during CAST, and total drop in T_rec_ during CAST ↑; T_sk_ ↓ and ↑ T_re –_ T_sk_ gradient; Larger ↑ in plasma NE
Bittel [97]	10 M	5 consecutive days/wk daily 1–3 h cold water immersion (to tolerance) over 2 months	10–15°C water; wearing neoprene diving suit	Standard cold test pre and post: 2 h supine on wire mesh bed wearing swimming trunks in 10°C air, wind speed 0.8 m/s, 40% RH	Post acclimation: ↑ in the delay for onset of shivering, ↓ T_b_ at onset of shivering, ↓ T_b_ in thermoneutrality, ↓ of heat debt by three mechanisms: (1) ↑ in metabolism without any variation of heat loss (n = 1), (2) ↓ heat loss without changes in metabolic heat production (n = 3), and (3) ↑ metabolic heat production associated with a ↓ heat loss (n = 5)
Golden [94]	16 M: 8 M passive immersion, 8 M exercising while immersed	10 head-out cold water immersions over 2 weeks: 2 shivering threshold immersions, 8 40 min resting cold water immersions	Shivering threshold immersion: 35–35.5°C water for 10 min, then water cooled by 1°C every 5 min Resting Immersions: 15°C, wearing only swimming trunks	Longitudinal passive observation: measurements taken throughout each individual immersion	In the static group: HR recorded over first 5 s of immersion and ventilation over the first 15s ↓ in last immersion than 1^st^, ↓ in initial shivering, ↑ initial thermal comfort (↓ in initial, first minute, responses to cold); ↓ metabolic response to prolonged immersion In the dynamic group: ↑ in metabolic response to cold in some subjects; ↓ VO_2_ during last shivering threshold immersion In both groups: ↔ T_c_ or T_b_
Stocks [95]	7 M	90 min passive cold-water immersions daily on days 2–7 and 9–14 (total 12 immersions), Immersed to the 4^th^ intercostal space seated	18.4°C; wearing only swimming costumes	Cold-water stress tests (CWST) on days 1, 8, 15: ~18°C 60 min seated + 30 min cycling	↓ in thermogenic response during the rest phase of the 3^rd^ CWST beyond 20 min compared to the 1^st^, extending only into the first 10 min of exercise; ↔ T_es_,T_sk_
Lunt [102]	32 M; 16 M cold water immersion, 16 M thermoneutral water immersion	6, 5 min water head-out immersions (2x/day)	Cold (12°C) or thermoneutral (35°C)	Pre and post testing: 100 W cycling breathing normoxic (F_IO2_ = 0.21) and hypoxic (F_IO2_ = 0.12) mixtures	Post repeated cold water immersion, ↑ HRV high frequency power and ↓ adrenaline and noradrenaline during hypoxic exercise exposure; Adrenaline and noradrenaline ↓ during hypoxic exercise after cold water immersion compared to thermoneutral immersion; ↓ in number of hypoxic symptoms and symptom severity with cold water immersion group but not thermoneutral group
Tipton [103]	21 M; split into 3 groups: CON (n = 7), CORE (n = 7), SKIN (n = 7)	CORE group: 5 head-out cold water immersions where T_rec_ was reduced by 1.18°C and T_sk_ decreased to 13.48°C SKIN group: 5 head-out cold water immersions for only 5 minutes, only T_sk_ was reduced to 13.52°C	CORE & SKIN groups: 12°C water wearing a bathing costume	Pre and post testing: 2 head-out immersions one-two weeks apart in stirred water at 12°C until rectal temperature fell to 35°C or 90 min had elapsed; wearing bathing costume	Only the deep-body cooling (CORE group) displayed a ↓ metabolic response during the post immersion until T_rec_ ↓ by 1.18°C, with no habituation observed when cooled further; SKIN group showed habituation in the ventilatory response during the first 5 min of the post immersion but ↔ in metabolic response; Overall, ↓ in skin and deep-body temperature can habituate the metabolic response with tissue temperature specificity, cooling of only skin temperature is sufficient to lower the cold shock response but not capable of inducing habituation of the metabolic response
Brazaitis [111]	14 M	17 total sessions (14 consecutive days) of head-out cold water immersion over 20 days; for session 1–16 cold water exposure until T_rec_ of 35.5°C or until 120 min of exposure, session 17 followed the same duration as session 1; for all sessions subjects were removed from cold immersion every 20 min for 10 min and then resumed	14°C cold water; wearing swimming shorts	Longitudinal passive (time-focused) observation; Cold water immersions 1 and 16 served as a pre and post test; immersion 17 served as an immersion 1 time matched post test	In first 6 sessions, a hypothermic acclimation (↓ metabolic heat production, VO2, shivering sensation, and T_rec_) was found that transitioned to hypothermic-insulative from sessions 7–16 marked by greater ↓ T_sk_ and T_rec_ with ↔ in metabolic heat production; the time-matched control (session 17) demonstrated a hypothermic acclimation marked by ↓ in metabolic heat production and greater ↓ in T_rec_ with ↔ T_sk_; presence of metabolic thermogenesis ↑ only present under thermoneutral conditions; ↓ cold-stress markers, activity of the innate immune system, suppression of specific immunity, and discomfort and cold sensation; in both sessions 16 & 17 ↓ in intramuscular temperature
Gordon [125]	7 M	1 h of daily head-out circulated cold water immersion for 7 consecutive days	14°C (designed to ↓ core temperature by ~1°C daily); wearing swimming trunks	Pre and post testing: Novel skin temperature clamping (26°C) cold exposure protocol using a liquid conditioned suit passively administered for 150 min	Acclimation protocol ↓ total shivering intensity by 36% with ↔ whole body heat production, suggesting non-shivering thermogenesis from skeletal muscle can be increased substantially by as little as 7 days of cold exposure; T_es_ daily rate of ↓ was reduced on average by ~0.01°C/min and thermal sensation ↑ from day 1 to 7

Radomski and Boutelier used an intermittent adaptation protocol of 9 immersions over 14 days in 15°C water for 25–40 min to pre-adapt participants before an Arctic excursion [93]. The pre-adapted group showed a blunted decrease in skin temperature during a 10°C cold air test prior to the excursion, while also demonstrating reduced norepinephrine (NE) excretion and cold sensations during 16 days in the Arctic. The authors reported fewer hormonal markers of stress in the pre-adapted group even though the groups experienced the same environment (−27°C) and performed the same tasks throughout the Arctic excursion, indicating that the pre-treatment had indeed caused a habituation of the cold stress response.

Golden and Tipton immersed participants for 40 min in 15°C water 10 times over 2 weeks and, when tested in the same condition, observed reductions in thermal sensation, HR, respiratory, and metabolic responses with a delay in shivering [94]. Stocks et al. immersed participants in 18°C water for 90 min for 15 straight days and similarly observed reductions in HR and metabolic responses [95]. Taken together, these short-term, laboratory-based, whole-body immersion studies indicate that thermoregulatory responses, particularly metabolic responses, exhibit habituation after repeated cold water exposures.

In a longer protocol, Young et al. had participants undergo 24 exposures to 18°C water for 90 min over 5 weeks and, when subsequently exposed to a 90 min 5°C cold air exposure, the investigators observed a larger drop in core and skin temperature and a slight delay in shivering, indicating an insulative-hypothermic adaptation [96]. In a similarly designed study, Bittel exposed participants to 10–15°C water for 1–3 h/day totaling 40 exposures over 8 weeks, observing unchanged or blunted core temperature responses, lower skin temperatures, and an increased overall metabolic response with a delay in shivering, indicating an insulative-hypermetabolic adaptation [97]. These findings suggest that habituated peripheral responses following short-term, repeated coldimmersions may develop into insulative and/or hypermetabolic adaptations with longer-term cold water exposure, though the habituated or blunted shivering response may persist.

### Short term water exposure: Habituation of the cold shock response

Other adaptations specific to cold water immersion relate to the reflex inspiratory gasp and subsequent cardiovascular (HR) and respiratory (tidal volume, breathing frequency) responses that comprise the CSR [55,56]. Habituation of the CSR has important implications for increasing chances of survival. Lessening the response can reduce the risk of water inhalation upon accidental immersion, thus decreasing the risk of drowning. There have been many studies on the topic, all of which consistently report habituation of the HR and ventilatory responses after neck deep immersion utilizing 5–7 repeated immersions of 2–7 min each in 10–15°C water over 1 to 9 days (Table 8) [98–105]. Partial immersions (half of body split down midline) [98], and repeated cold showers [106] have also been shown to reduce some components of the CSR, the mechanistic implications of which will be expanded upon below.

**Table 8.** Cold shock response studies.

Reference	Study Sample	Habituation Length	Habituation Temperature	Cold Testing Procedure	Results/Findings
Tipton [98]	11 M, 4 F; 8 in habituation group, 7 CON	6, 3 min head-out immersions in stirred cold water of the left side of the body over 3 days (2x/day)	10°C water; wearing swimming costume and halved wetsuit for non-immersed side	Pre and post testing: 3 min head-out immersions in stirred water at 10°C of the right side of the body	Repeated (6) left-side immersions ↓ the magnitude of HR, respiratory rate and volume responses during the 2^nd^ right-side immersion in the habituation group without any change in T_sk_
Tipton [100]	12 M; 8 in habituation group, 4 CON	6, 3 min head-out immersions over 3 days (2x/day)	15°C water; wearing swimming trunks	Pre and post (immediately following the completion of the 6 repeated immersions and again at 2, 4, 7, and 14 months) testing; 3 min head-out seated immersions in stirred water at 10°C wearing swim trunks	Habituated subjects: ↓ respiratory frequency, inspiratory minute volume, and HR during the 1^st^ 30 sec of immersion immediately post repeated immersion (retained for 7 months); After 14 months, HR remained ↓ but respiratory frequency and inspiratory minute volume returned to near pre-habituation levels; Periodic immersions incurred by the CON group (as well as the greater volume of immersion in the habituation group) ↓ the duration of reactive elevation in HR, tidal volume, and inspired minute ventilation
Eglin [106]	13 M, 5 F	6 cold showers over 3 days (2x/day); 3 exposure groups: (1) 3 min at 10°C on the back (10B), (2) 3 min at 15°C on the back (15B), (3) 30 sec at 10°C on the back + 30 sec on the front (10BF)	10°C 15°C 10°C	Pre and post testing: 3 min head-out immersions in stirred water at 10°C wearing swim wear	Over first 30 sec of immersion, immersion respiratory frequency was ↓ by 21% in groups 10B and 10BF after repeated showers, but not 15B; the rate of change of skin temperature is an important factor in determining the degree of respiratory drive habituation
Barwood [101]	20 M; 10 M habituation, 10 M habituation + psychological skills training	5, 2.5 min head-out cold water immersions (2x/day) breathing freely	~12°C; wearing swimming trunks	Pre and post testing: 2.5 min seated, head-out immersions in stirred cold water (~12°C) wearing swimming trunks while maximally breath holding	Following repeated immersions, both habituation and habituation + psychological skills training ↑ breath holding time (by 73% and 120%, respectively), ↓ HR at 2 min of cold water immersion, and ↓ breathing frequency throughout cold water immersion
Barwood [110]	8 M, 4 F	7, 7 min head-out immersions (1x daily for 7 days); Immersions 1 and 7 were cold water (CWI) and immersions 2–6 were thermoneutral water (TWI)	CWI: 15°C TWI: 35°C Wearing a swimming costume	Longitudinal passive (time-focused) observation; Cold water immersions 1 and 7 served as a pre and post test	↓ in self-reported acute anxiety from CWI 1 to CWI 7 but ↔ in HR, breathing frequency, or minute ventilation as part of the cold shock response; Tidal volume ↓ from CWI 1 to CWI 7
Eglin [104]	9 M	5, 3 min head-out immersions into cold water over the course of a collective 55–120 min; rewarmed in 38°C for 3 minutes + 10 minute break between cold water immersions	15°C water; wearing swimming trunks	Pre and post testing: 2 head-out immersions into 15°C stirred cold water for 5 minutes wearing swimming trunks (IMM1 and IMM7); one week apart	HR ↓ throughout IMM7 compared to IMM1; inspiratory minute volume ↓ IMM7 compared to IMM1 over the 1^st^ minute of immersion; respiratory frequency ↓ in the first 30 sec in IMM7 vs. IMM1; ↔ in inspiratory gasp and tidal volume
Barwood [105]	Group (1): 12 M, 4 F Group (2): 6 M, 4 F	7, 7 min head-out cold water immersions (1x daily for 7 days); Two experimental groups: (1) Repeated anxiety, where anxiety was raised for each immersion using deception and math tasks and (2) Acute anxiety, where deception was only used once for the 1^st^ immersion	15°C water; wearing swimming costume	Longitudinal passive (time-focused) observation; Cold water immersions 1 and 7 served as a pre and post test	↔ in anxiety levels between immersions 1 (pre-control), 7 (post-control), and mean of habituation immersions for repeated anxiety group; Repeated anxiety during habituation resulted in failure of a habituation of the cold shock response even when additional anxiety was removed (↔ HR, respiratory frequency, tidal volume, or minute ventilation)

## Timeline of cold habituation

The methods of cold habituation studies vary greatly, with laboratory acclimation periods ranging from 5–80 repeated exposures over 1 day up to 2 months, to studies of those who have experienced natural lifetime cold exposure. Cold habituation itself involves several factors, as previously mentioned, all with varying timelines of adaptation. Habituation of cold sensation appears to be the first to occur, typically showing reduced ratings after the 1^st^ or 2^nd^ exposure [85,107–109]. Pain from intense cold applications/exposures (cold pressor, thermodes) also start to habituate by the 2^nd^ trial [85] with others reporting significant decreases in pain by the 5^th^ day of repeated exposures [83]. The anxiety associated with cold water immersions has been shown to be reduced by about the 3^rd^ day of repeated immersions [110], which may be a factor in the habituated cardiorespiratory responses related to cold shock, as these responses are significantly reduced by the 4^th^ or 5^th^ repeated immersion [98–101]. Perhaps surprisingly, habituation of the CSR may persist for several months after habituated responses are achieved, with some components remaining habituated for up to 14 months [100], indicative of a long-term or long-lasting habituation. However, the decay of CSR and other cold habituated responses remains largely unexplored.

During cold air exposures, vasoconstriction and BP responses seem to have variable timelines of habituation. Leppaluoto et al. showed habituated vasoconstrictor and BP responses at different times between days 4–8, but by the end of the 11 day exposure, these responses had returned to a non-habituated state, despite circulating NE, a marker of sympathetic activity, being lower on both days 5 and 10 [107]. The delay in shivering reported by Bruck and colleagues occurred by the 3^rd^ exposure [66]. Brazaitis et al. reported decreases in shivering, metabolic heat production, and cold discomfort during the first 6 immersions. After the 6^th^ immersion, they reported a further decrease in shivering, yet showed an increase in metabolic heat production, perhaps indicating that the 6^th^ to 7^th^ immersion may be the threshold point for the shift from shivering-derived to non-shivering-derived thermogenesis [111].

**Figure 3.**
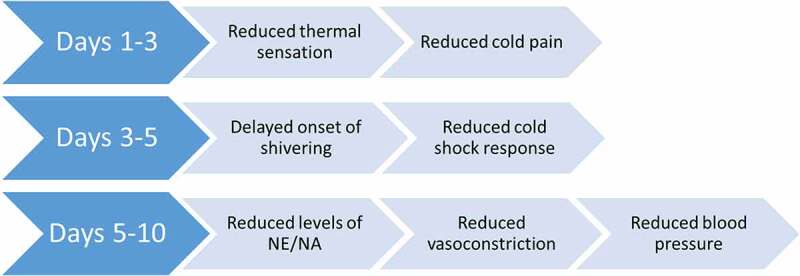
Timeline of changes in perceptual and physiological responses due to cold habituation.

While time course data remain limited, the current literature suggests that cold habituation responses likely occur between the 3^rd^ and 11^th^ exposure. As exposures become more severe (colder temperatures, longer durations, water immersion), physiological changes may advance into systemic insulative or hypermetabolic adaptations rather than habituated responses, as seen during prolonged repeated (1–3h/day, 3–5 weeks, 10–20°C) cold water immersions [96,97,111]. Figure 3 summarizes the time course of perceptual and physiological changes due to cold habituation.

## Specificity of cold habituation

Habituation to the cold is often reported when an individual is repeatedly exposed to a cold stimulus, with pre- and post-acclimation measurements occurring in the same environment. For example, in the cold air tests listed above, participants experienced the same exposure each day which eventually resulted in a habituation of the physiological responses to that specific cold stimulus. Many studies that fail to see habituation tend to test for adaptations using a different type of cold stimulus (e.g., cold water vs cold air or local vs whole-body). In the aforementioned study by Savourey et al. for example, one month of twice-daily thigh-deep cold water immersions induced habituated responses (i.e., higher skin temperatures and a smaller rise in BP) during a standardized 5°C foot immersion [79,80]. Conversely, during a 1°C whole-body cold air exposure of those same participants, lower skin and core temperatures were observed (with no change in metabolic heat production), in what appeared to be a hypothermic-insulative type of adaptation. These data suggest that habituated responses may be specific to the acclimation stimulus.

Temperature and severity of the cold exposure also play a role in the level of adaptation observed. Habituation of the initial CSR to a particular water temperature can be induced, at least to a degree, with repeated exposures to a milder stimulus. Tipton et al. exposed participants to six 3-min head out immersions in 15°C water over 3 days and reported partially habituated responses of respiratory frequency, minute volume, and HR when the participants were immersed in 10°C water [99]. Similarly, a study by Eglin and Tipton used cold showers in an attempt to habituate the CSR upon chest-deep immersion [106]. Participants were showered with 10–15°C water on small body surface areas, either the chest, back, or both for 1–3 min totaling 6 exposures over 3 days and were then immersed in 10°C water. Although only a portion of each participant’s body was directly repeatedly exposed to cold, a partial reduction in CSR was observed upon whole body exposure.

However, in a different study, these authors reported an adaptation threshold when they immersed participants in 12°C water for different lengths of time [103]. One group was cooled until skin temperature dropped to 13.5°C (skin cooling only) while the other was cooled until core temperature dropped by 1.2°C (skin and core cooling). During the test exposure, both groups showed habituation of the immediate CSR. However, as the skin cooling group’s exposure began to exceed their previous level of cold stress, physiological responses returned to a non-habituated level. The core cooling group continued to show a habituated metabolic response until their core cooled past the level that was previously experienced during the repeated exposures, after which it returned to a non-habituated state – that is, the metabolic heat production response was no longer blunted.

In a study examining the perceptual responses to local cold water immersion, Carman and Knight performed a test using foot exposures to colder water than the repeated exposures, and reported a similar adaptation threshold [83]. Participants were either repeatedly exposed to 1°C or 5°C, and were then tested in the opposite condition. Those exposed to a lower temperature than previously experienced showed pain responses that returned to baseline, rather than remaining habituated. Together, these studies indicate a specificity of the habituation response, where a change in the type or severity of exposure often results in responses returning to a non-habituated state.

## Mechanisms of cold habituation

Multiple potential mechanisms may contribute to blunted thermoeffector responses following cold habituation and include reduced sensory input from skin thermoreceptors, altered central processing of afferent signals and autonomic activation, and/or altered peripheral mechanisms. As mentioned previously, rodents and cats housed in 5°C show decreases in the sensitivity of peripheral thermoreceptors responsive to low temperatures [22]. In humans, there are data, though limited, suggesting that the number of functioning cold receptors may be reduced in the forearm skin of occupational workers routinely exposed to cold air in the winter months, and this reduction in estimated functioning receptors has been associated with reduced thermal sensation [22]. Humans frequently report lower cold sensation and pain ratings after repeated cold exposures, potentially indicating a role for similar peripheral sensory mechanisms.

One of the interesting questions concerning cold habituation is whether habituated cold responses cross over to non-cold exposed sites, as it may indicate a largely central mechanism contributing to habituation. Carman and Knight repeatedly exposed one foot of participants to 5°C for a total of 40 exposures, resulting in a habituation to cold pain [83]. Leblanc and Potvin showed similar results when exposing participants’ left hands to 40 repeated cold water immersions, resulting in reduced pain responses [81]. In both studies, when testing the contralateral body part in the same temperature used for habituation, pain responses were either similar to pretest levels or were sensitized (i.e. augmented response for a given stimulus), suggesting that only the repeatedly exposed part had habituated, potentially supporting a peripheral, rather than central, mechanism. Interestingly, when Eagen performed repeated exposures of just the middle finger, the immersed finger and not the contralateral finger had reduced cold pain, yet the reduced drop in finger temperatures while immersed was similar [78]. This may indicate that cold perception is location specific, while other vascular or physiological adaptations may be more contralaterally transferable.

In line with this notion, Tipton et al. reported that habituated CSR can indeed cross over to the opposite half of the body [98]. Using a specially designed wet-suit for half-body exposures, subjects were exposed only on the left side of the body to six 3-min immersions in 10°C water over a 3-day period. Post-testing on the right-side of the body revealed a habituation of the CSR. This habituation of non-cold exposed regions points to a central mechanism for habituation and suggests that habituation of the CSR is not reliant on direct peripheral stimulation [98].

Additional evidence for altered central integration of afferent signals or autonomic activation is provided by multiple human studies that include measures of circulating catecholamines. Reductions in plasma NE concentrations have been consistently observed following short-term, repeated cold air exposures [6–8], suggesting that cold habituation lowers sympathetic activation. It is worth noting that these studies also demonstrated that habituated responses to cold air occur independent of any change in thyroid stimulating hormone or thyroxine, two hormones that are known to regulate basal metabolism.

More direct study of brain activity provides further evidence of habituation occurring through a central mechanism in humans. Mulders et al. subjected volunteers to sinusoidal cooling (between 31 and 14°C) on the forearm and measured perception ratings over time alongside electroencephalogram (EEG) activity [109]. They found that perception of cooling was reduced over time and EEG activity was attenuated. The authors theorized that changes in peripheral sensation reduced brain activity to the cooling stimulus which may have been caused by receptor fatigue or a peripheral adaptation. Involvement of specific brain regions was also studied by Klingner et al. The authors used blood-oxygenation dependent level signaling as a measure of neuronal activity and showed a decrease in deoxyhemoglobin concentration in the somatosensory cortex following repeated median nerve stimulation that led to habituation, indicating decreased neuronal input and local processing in the parietal lobe [112]. The frontal areas of the cerebral cortex have also been suggested to be needed to develop habituation; data from rats indicate that bilateral frontal lesions prevent habituation of the HR response [113]. Data from humans using chlorpromazine [114] further suggest the frontal cortex area is important for habituation. However the mechanism for this habituation is not known since chlorpromazine blocks cholinergic, adrenergic, dopaminergic, and histamine receptors. Although little is known about the pathways involved in habituation of the cold response, mechanisms of habituation are often located centrally, either occurring in the spinal cord or in higher brain systems.

### Cold-induced vasodilation

Humans who regularly experience occupational local cold water exposure, such as Gaspe fishermen [72] and British fish filleters [74], and/or are habitually exposed to systemic cold, such as the Eskimos [57] and Lapps [115], exhibit a habituation response marked by greater hand skin temperatures that are often explained by an earlier occurrence of CIVD when exposed to cold water. The meaningful induction of this habituation response during incidental and/or intentional systemic cold exposure has been sought after by interested military personnel from both cold and temperate climates alike.

Livingstone et al. [116,117] performed a series of investigations involving CIVD modulation amongst Canadian Armed Forces Personnel following 14–91 days of Arctic temperatures. Post-Arctic exposure, personnel underwent 30 min of finger ice water (0–0.1°C) exposure to evaluate CIVD responses. Interestingly, 14 days of exposure elicited lower initial finger temperatures than before testing, an increase in the time to first temperature rise, a decrease in the first vasodilative rise temperature, and a decreased average finger temperature during 5–30 min of ice water immersion. In contrast, 91 days of similar Arctic exposure elicited the opposite response in all three variables: decreased time to first temperature rise, an increase in the first vasodilative rise temperature, and increased average finger temperature during 5–30 min of ice water immersion. While length of exposure may at least partially explain the differential response, later work by Livingstone et al. suggests that the influence of seasonal cold exposure in Canada may prove to be just as impactful to CIVD as Arctic exposures and therefore confound full CIVD modulation interpretation [118].

Branching away from personnel that may regularly experience cold in their daily lives, seasonal or otherwise, further investigations sought to test the ability of 28 days of systemic Arctic (−37 to −12°C outdoors, 7–20°C inside sleeping huts) cold exposure to influence CIVD in tropical Indian soldiers as compared to temperate Russians and Arctic natives. CIVD was evaluated using whole-hand immersion to the styloid process at 4°C for 30 minutes. Twenty-eight days of exposure was sufficient to increase mean skin temperature, finger blood flow, and a CIVD index in the tropical Indians to a level that was similar to the migrant Russians but still lower than that of the Arctic natives. While no definitive relation of CIVD response to oral temperature was observed, oral temperature tended to show a slight fall or rise for very good or very poor CIVD responders, respectively [119]. Other published work from these same authors further suggests that these improvements in CIVD following repeated cold exposure may be accompanied by a reduced sympathetic response as indicated by a reduction in cold pressor reactive BP and HR [120].

Collectively, the case can be made that a foundational ability to undergo CIVD fluctuations following systemic cold exposure persists within humans but is sympathetically clamped to a degree proportional to the combined influence of the level of cold stress as well as the degree of hypothermia. It might be said that the level of cold stress is influenced by the novelty, severity, acute cold exposure volume (How long have the individuals been exposed to a cold stimulus recently?), and chronic cold exposure volume (consistent systemic cold exposure over their lifetime vs. seasonal systemic cold exposure vs. no consistent or seasonal systemic cold exposure). The complex interplay between these factors does not currently allow for clear determination of the degree to which repeated cold exposure may predictively modify CIVD responses and variations in CIVD methodologies contribute to difficulties in precise comparison of study-to-study results [29].

### Non-shivering thermogenesis

Changes in thermogenesis in brown adipose and muscle tissue have recently been examined following repeated days of cold exposure [121–123]. Interestingly, repeated cold exposures may increase NST in the presence of a habituated shivering response. Blondin et al. exposed individuals to 20 cold exposures, via a water perfused suit (10°C), over a 4 week period. Pre- and post-cold acclimation testing was conducted during a 4°C stimulus in the water-perfused suit. Following the 4 weeks of repeated cold exposure, they observed a 21% overall reduction in shivering intensity but no change in total heat production [45,122], suggesting that NST increased. Interestingly, they found that: a) BAT oxidative capacity increased by 45% when subjected to 18°C water in the suit, b) the coupling between muscle activity and metabolic heat production was improved, c) the proton leak in muscle that occurs with acute cold exposure was abated (suggestive of an increased coupling of oxidative phosphorylation), and d) continuous and burst shivering was lowered in the *vastus lateralis*. Their data point to a shift in NST from skeletal muscle to BAT. Furthermore, these authors [123] have found that repeated, relatively mild cold exposure (water-perfused suit at 10°C) decreases deep muscle recruitment that has been shown to occur during acute cold exposure [124]. Indeed, in their repeated cold exposure study, [18 F]fluorodeoxyglucose, a marker of shivering activity, decreased in the *longus colli* and *sternocleidomastoid*.

In a similar but shorter adaptation protocol consisting of just 7 days of cold water immersion, reductions in shivering thermogenesis were observed during a cold test in which skin temperature was clamped at 26°C using a water-perfused suit. Total heat production was the same before and after the 7 days of cold exposure, indicating that there may have been a shift from shivering to NST [125]. These results indicate that shivering, but not overall heat production, became habituated. The authors postulated that a potential mechanism for NST in muscle cells may occur through an uncoupling mechanism, which is likely activated by the binding of the peptide sarcolipin to SERCA, the Ca^2+^ pump located in the sarcoplasmic reticulum membrane, causing Ca^2+^-slippage and generation of heat [126], although this has yet to be confirmed. It is important to note that this shift to NST may require a more severe or prolonged period of cold exposure and/or may represent a later phase of cold adaptation that follows the initial reduction in total metabolic heat production often associated with habituation.

## Modifiers of cold habituation

Low temperatures are often encountered together with hypoxia (i.e., at high-altitude); thus, the effect of concurrent hypoxic exposure on cold habituation is particularly relevant to field environments. Given that hypoxic exposure can modulate acute and adaptive responses to cold [127–130], adaptation to cold may be altered at altitude. In an effort to answer this question, Keramidas and colleagues examined a group of individuals on an 11-day expedition on the Antarctic Plateau, a high-altitude region with low ambient temperatures [131]. Following the expedition, the authors observed a hypothermic pattern of cold acclimation characterized by attenuated cutaneous vasoconstriction and suppressed shivering, which suggests that normal habituation responses to cold occur during concomitant hypoxic exposure at altitude.

Other concurrent stressors, including mental stress, fatigue, sleep deprivation, and caloric restriction also alter physiological responses to cold stress [2,132] and thus may modify the cold habituation response. LeBlanc and Potvin used mental arithmetic to stress participants during 40 repeated cold water hand immersions (2.5 min, 4°C) over 4 weeks [81]. Group 1 was exposed to cold water only, while Group 2 was required to add numbers verbalized by the experimenter during each hand immersion. Both groups showed reduced BP responses over time, but when mental arithmetic was removed and Group 2 was only exposed to the cold water test, perceptual and pressor responses returned to a non-habituated state. The authors suggested that the added mental stress distracted from the cold exposures and inhibited habituation to cold alone. The authors also saw this as evidence for a central mechanism to habituation, as no peripheral habituation occurred during repeated exposures in the combined stressor group, and perhaps indicates an interconnectedness of learning pathways that could not be separated thereafter.

More recent data from Barwood and colleagues demonstrated that when high levels of anxiety are superimposed on whole-body cold water immersion, habituation of the acute cardiorespiratory response (i.e., CSR) to cold water immersion is blunted [105,133]. Moreover, acute anxiety partially reverses habituated responses to cold water immersion in already habituated individuals [133]. Brain regions in the frontal and prefrontal cortex that respond to thermal afferent information and are likely involved in habituation [113,114] are shared with those that respond to certain psychological responses and therefore may be a central location by which anxiety modulates cold-water habituation. Specific alterations in common neural circuitry that mediate the effects of anxiety on cold habituation remain speculative but provide an important line of inquiry. Examining the effects of mental stress on adaptation responses to cold exposure is particularly relevant for occupational groups and military personnel, as they often encounter such combined stressors in operational field environments. Survival training that targets lowering anxiety before and during immersion may provide a protective benefit for at-risk personnel.

Habituation may be modified by a psychological component. Smith et al. provided evidence showing that those with higher self-reported resiliency and life purpose ratings were able to better habituate to repeated cold pain, which suggested that motivation levels may affect an individual’s willingness to endure the cold for long enough time-periods to allow habituation to occur to a greater degree [85]. Recently, Park and Lee provided evidence supporting this idea by showing that those who self-identified as having a high tolerance to cold had stronger CIVD responses than those with self-identified low tolerance [134]. This raises the question as to whether psychological components can have relevant effects on physiology or if those who naturally display strong physiological attributes also naturally have higher psychological resolve.

Sleep deficiency has also been shown to prevent or delay habituation responses [84,135,136]. Although the effects of sleep restriction on habituation of physiological cold responses remain largely unexplored, sleep-deprived individuals appear to be less able to habituate to pain associated with cold-water hand immersion (i.e., cold pressor test), which may be due to alterations in central pain-inhibitory signaling pathways [84]. However, the potential effects of sleep deprivation, along with fatigue and caloric restriction, on vascular, metabolic, and cardiorespiratory responses to cold exposure require further investigation.

## Cross-adaptation: Cold habituation and altitude

As discussed above, the primary responses to cold exposure, including peripheral vasoconstriction and increased metabolic heat production (i.e., shivering), are driven by activation of the autonomic nervous system [2,4]. It is well-established that cold habituation blunts the sympathetic response to cold exposure, but evidence also suggests that cold habituation alters autonomic responses to altitude, which may have important metabolic implications for hypoxic exercise [102]. This phenomenon whereby an environmental exposure provides physiological adaptations to an alternative environment is referred to as cross-adaptation.

In humans, attenuated metabolic responses associated with cold habituation have been linked to improvements in exercise economy in the cold [i.e. lower oxygen uptake and respiratory exchange ratio (RER)] [137]. A lower RER is reflective of preferential fat oxidation, which may provide a beneficial cross-adaptation effect at altitude if carbohydrate utilization is spared for exercise performance. In line with this notion, there is evidence, albeit limited, to suggest that alterations in the autonomic and metabolic responses following repeated cold exposure may confer performance benefits at altitude. In a study by Lunt et al., repeated cold-water immersions (12°C) reduced the sympathetic response to acute hypoxic cycling exercise, evidenced by reduced circulating catecholamines and increased high frequency power of HR variability [102]. In addition, ventilation, oxygen uptake, and RER were reduced and symptom severity responses to hypoxia were improved following the cold-water immersion intervention. These data suggest that a habituation response to cold may provide a performance benefit for individuals that perform physical work at altitude. However, the 10-min exercise bout in this study was short in duration and was performed in normobaric hypoxia, limiting its generalizability to prolonged exercise performance at altitude. Additional studies are required to further investigate the functional effects of cold habituation on responses to altitude and the mechanisms mediating a potential cold-to-hypoxia cross-adaptation. While there are multiple mechanistic pathways and proteins (e.g., cold-induced RNA binding protein and shock proteins) that are responsive to both cold and hypoxia, the specific cellular responses that may contribute to this cross-adaptation have not been examined.

## Future directions

Despite the abundance of cold research, there remains a multitude of unanswered questions within the literature. Firstly, the physiological mechanisms responsible for cold habituation remain unclear. There is evidence for both peripheral and central factors in habituation of vasoconstriction, shivering, perception, and CIVD. Determining the specific mechanisms (e.g., neural or vascular) for each of these responses will greatly expand our understanding of the physiological and psychological changes that occur with repeated cold exposure. Along with the mechanisms, the timeline of change for each of these factors is ambiguous. Comparing results of various studies gives insight into the potential timeframe of change, but more controlled experiments with continuous measurements are needed to construct a more precise timeline, along with long-term follow-up to characterize its decay. Parsing out the most efficient exposure type, length, and severity will be important in determining how to optimize the development of cold habituation, which could potentially translate into cold exposure regimens for outdoor workers and athletes (elite and recreational) to improve their physiological and psychological performance. Furthermore, there is limited evidence that cold habituation may change responses to other environmental stressors. Exploring this in detail may lead to exciting new discoveries on cross-adaptation and physiological mechanisms that are shared by different extreme environmental exposures.

## Summary and conclusions

**Figure 4.**
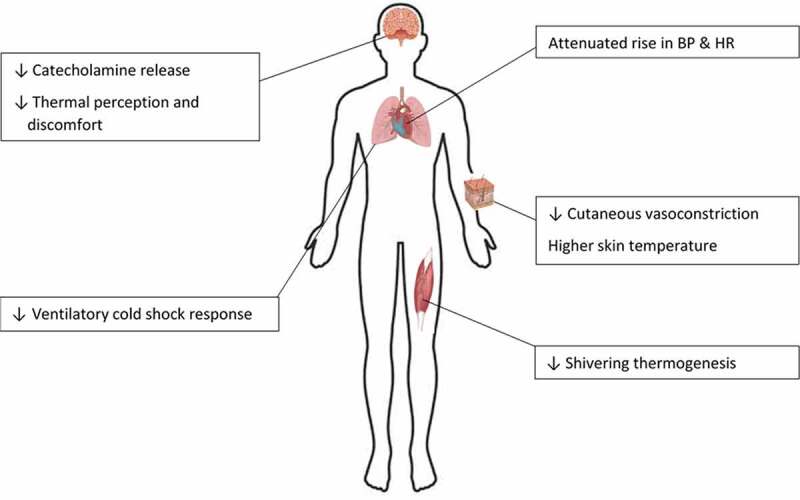
Summary of the physiological and perceptual changes that occur due to cold habituation in humans.

Habituation is a process of learning in which repeated exposure to a stimulus leads to decreased behavioral and neuronal responses [14,138], the purpose of which is to distinguish vital information from extraneous noise. The habituation process is evolutionarily conserved from one-celled organisms to mammals and occurs in response to various forms of repeated stimuli. In humans, cold habituation is marked by a blunting or attenuation of the thermoeffector responses of peripheral vasoconstriction and shivering thermogenesis as well as perception of cold (Figure 4). Habituated cold response mechanisms are demonstrated after repeated short duration mild cold exposures and may benefit the human through increased skin temperatures and decreased shivering. Cold habituation also results in decreased sympathetic nervous system activation, the effects of which may offer benefits in other environments such as high altitude. Based on current literature, habituation seems to occur mainly through central mechanisms, but some aspects may be mediated by mechanisms in the periphery. Further research is needed to fully parse out habituation-driven mechanisms in an effort to determine the most efficient means to induce habituation in benefit of the cold-exposed human outdoors.

**Figure:**
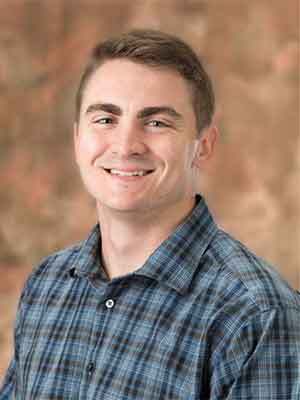

**Figure:**
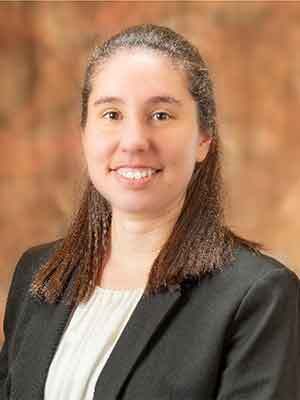

**Figure:**
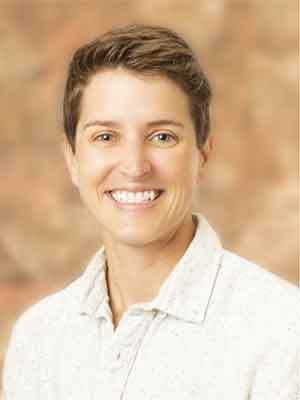

**Figure:**
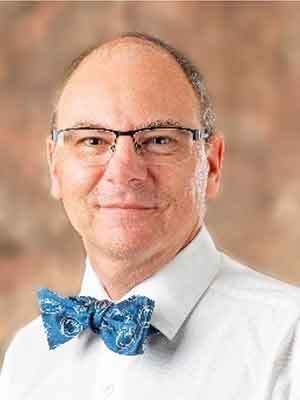

## Abbreviations

HPA = hypothalamic-pituitary axis
SNS = sympathetic nervous system
DEG-1 = degenerin/epithelial Na+ channel
CIVD = cold-induced vasodilation
NST = Non-shivering thermogenesis
BAT = brown adipose tissue
MAP = mean arterial pressure
CSR = cold shock response
HR = heart rate
BP = blood pressure
NE = norepinephrine
EEG = electroencephalogram
RER = respiratory exchange ratio

## Disclaimers

The views, opinions, and/or findings contained in this article are those of the authors and should not be construed as an official United States Department of the Army position, or decision, unless so designated by other official documentation. This article is approved for public release, and distribution is unlimited.

Citations of commercial organizations and trade names in this report do not constitute an official Department of the Army endorsement or approval of the products or services of these organizations.

This research was supported in part by an appointment to the Department of Defense (DOD) Research Participation Program administered by the Oak Ridge Institute for Science and Education (ORISE) through an interagency agreement between the U.S. Department of Energy (DOE) and the DOD. ORISE is managed by ORAU under DOE contract number DE-SC0014664. All opinions expressed in this paper are the author’s and do not necessarily reflect the policies and views of the U.S. Army, DOD, DOE, or ORAU/ORISE.
